# Acute Toxicity and Determination of the Active Constituents of Aqueous Extract of *Uncaria tomentosa* Bark in *Hyphessobrycon eques*

**DOI:** 10.1155/2014/412437

**Published:** 2014-03-06

**Authors:** Jefferson Yunis Aguinaga, Gustavo S. Claudiano, Paulo F. Marcusso, Cynthia Ikefuti, George G. Ortega, Silas F. Eto, Claudinei da Cruz, Juliet R. E. Moraes, Flávio R. Moraes, João B. K. Fernandes

**Affiliations:** ^1^Aquaculture Center of UNESP, Jaboticabal, SP, Brazil; ^2^Department of Veterinary Pathology, School of Agrarian and Veterinary Sciences, São Paulo State University (UNESP), 14884-900 Jaboticabal, SP, Brazil; ^3^Weed Science Environmental Research Studies Centre of UNESP, University Estadual Paulista, Jaboticabal, SP, Brazil; ^4^Faculty of Pharmacy, Federal University of Rio Grande do Sul (UFRGS), Porto Alegre, RS, Brazil

## Abstract

*Uncaria tomentosa* is a medicinal plant used in folk medicine by Amazon tribes. In this study the constituents of aqueous extract of *U. tomentosa* bark were quantified by chromatographic technique and its lethal concentration 50 (48 h) in *Hyphessobrycon eques* was determined. The chromatography showed high levels of oxindole alkaloids, quinovic acid glycosides, and low molecular weight polyphenols. The CL50 48 h was 1816 mg/L. Fish showed behavior changes at concentrations above 2000 mg/L, accompanied by a significant decrease of dissolved oxygen. At the highest concentration 100% mortality was observed attributed to oxygen reduction by the amount of oxindole alkaloids, polyphenols accumulation of the extract in the gills, and the interaction of these compounds with dopamine. In conclusion, the aqueous extract of *U. tomentosa* did not alter the chemical components and it was shown that *U. tomentosa* has low toxicity to *H. eques*; therefore, it can be used safely in this species.

## 1. Introduction

*Uncaria tomentosa*, popularly known as cat's claw, is a plant of the Amazon forest belonging to the Rubiaceae family. It has been used by native people of the Peruvian rainforest in therapies against inflammations, cancer, viral diseases, and gastric ulcers [1]. Different studies have shown the presence of oxindole alkaloids, quinovic acid glycosides, and low molecular weight polyphenols in all parts of the plant. These compounds have immunostimulant, antiviral, anti-inflammatory, and antileukemic activity [2].

In the last years, the intensification of fish farming increased the occurrence of parasites and infectious diseases. In order to improve this situation, producers use large amounts of antibiotics and other substances that cause microorganism resistance, presence of residues in fish tissues, trade restrictions, and negative environmental impact [3].

In an attempt to reduce and avoid the use of synthetic drugs and their adverse effects, the use of herbal medicine is an alternative. Therefore, a chemical analysis of the herb is necessary to identify and quantify the active constituents. Moreover, it is essential to evaluate the toxicity of the herb for therapeutic purposes, because some products have therapeutic concentrations near to their lethal dose [4].

*Hyphessobrycon eques*, popularly known as Jewel tetra, is a neotropical fish widely distributed in South America. In recent years, it has been widely used in toxicity tests in Brazil, due to its condition as a local fish, adapted to environments that occur naturally in tropical South America such as high temperatures, acid, and soft water. Furthermore, this fish is quite prolific, shows high metabolism, and is highly sensitive to different substances, essential conditions for toxicological studies.

The aim of this study was to use the method of high performance liquid chromatography with photodiode array detector (HPLC-PDA) to assess and quantify the chemical composition of the aqueous extract of *U. tomentosa* bark and determine their median lethal concentration for Jewel tetra.

## 2. Materials and Methods

### 2.1. Plant Material and Extraction

A commercial extract of *U. tomentosa* bark derived from Peru was used in this study (“Casa Massaro,” S.A. of Brazil; cod. 0442) and directly shipped to our laboratory. The extract was powdered, spray-dried (SD-05, Labplant, UK), and stored in plastic bags. To prepare a stock solution (10%), the extract was suspended in deionized water for 24 hours (10 mg/mL). Afterwards, the stock solution was filtered (0.14 mm in Qualy porosity) and used in the biological test.

### 2.2. Assay of Polyphenols, Oxindole Alkaloids, and Quinovic Acid Glycosides on Plant Material

#### 2.2.1. LC Analysis of Low Molecular Weight Polyphenols

The analyses were performed employing an HPLC-PDA Proeminence (Shimadzu, Tokyo, Japan) by method previously validated [5]. Briefly, a Gemini RP-18 column (250 × 4.6 mm i.d., 5 *μ*m) (Phenomenex, USA) protected by a RP-18 guard column was used. The mobile phase consisted of 0.1% v/v trifluoroacetic acid (A) and methanol: TFA (99.9 : 0.1, v/v) (B) in a linear gradient program. The flow rate and temperature were kept constant at 0.9 mL/min and 23 ± 1°C, respectively, detection being performed at 325 nm. The total content was calculated by sum of individual contents, namely, chlorogenic acid (CLA), caffeic acid (CFA), unknown peaks (expressed as chlorogenic acid), and rutin (RUT). The results were expressed as *μ*g/mL of extractive solution by mean value of three determinations using the chlorogenic acid (Fluka, batch 455159/1, Switzerland), caffeic acid (Extrasynthèse, batch 0381024, France), and rutin (Sigma, batch 128K1177, USA) as external standards.

#### 2.2.2. LC Analysis of Oxindole Alkaloids

The analyses were performed employing a previously validated HPLC-PDA method [6]. Briefly, a Gemini-NX RP-18 column (250 × 4.6 mm i.d., 5 *μ*m) (Phenomenex, USA) protected by a RP-18 guard column was used. The mobile phase consisted of ammonium acetate buffer 10 mM (pH 7.0) (A) and acetonitrile (B) in a linear gradient program. The flow rate and temperature were kept constant at 1.0 mL/min and 23 ± 1°C, respectively, the detection being performed at 245 nm. The total pentacyclic oxindole alkaloids (POA) and tetracyclic oxindole alkaloids (TOA) contents were calculated by sum of individual alkaloid contents, namely, speciophylline, uncarine F, mitraphylline, isomitraphylline, pteropodine, and isopteropodine for POA and rhyncophylline and isorhyncophylline for TOA. The results were expressed as *μ*g/mL of extractive solution by mean value of three determinations using the mitraphylline (Phytolab, batch 2946, Germany) as external standard.

#### 2.2.3. LC Analysis of Quinovic Acid Glycosides

The analyses were performed employing a previously validated HPLC-PDA method [7]. Briefly, a Sinergy Fusion RP-18 column (150 × 3.9 mm i.d., 5 *μ*m) (Phenomenex, USA) protected by a RP-18 guard column was used. The mobile phase consisted of formic acid 0.01% (v/v) (A) and acetonitrile: formic acid 0.01% (90 : 10, v/v) (B) in a linear gradient program. The flow rate and temperature were kept constant at 1.0 mL/min and 35 ± 1°C, respectively, the detection being performed at 245 nm. The total quinovic acid glycosides content was calculated by the sum of individual contents of the seven major peaks previously characterized as quinovic acid glycosides through UV and MS-MS data [7]. The results were expressed as *μ*g/mL of extractive solution by mean value of three determinations using the *α*-hederin (Extrasynthèse, batch 08040314, France) as external standard.

### 2.3. LC50 Determination

The exploratory range of concentration of aqueous extract was determined with a series of range finding experiments. 108 Jewel tetra (*H. eques*) (1.76 ± 0.6 g) were acclimated in 250 L plastic box for 14 days. During this period, fish were fed with commercial diet (3% biomass, 28% crude protein, and 4000 kcal/kg) and subjected to a prophylactic bath of noniodinated sodium chloride (1%) for 60 min (Ethics Committees n° 003868/10).

In preliminary tests, fish were randomly distributed in 3 L glass tanks and exposed to 0.0, 1.0, 3.0, 5.0, 7.0, 10.0, 12.0, 15.0, 20.0, 23.0, 25.0, 50.0, and 75.0 mL of a stock solution during 48 hours. The intervals obtained were used for assembling the definitive test [8].

The 48 h LC50 final test was performed using 54 fish (1.86 ± 0.1 g) randomly distributed in 18 3 L tanks with concentrations of 0.0, 14.0, 16.0, 18.0, 20.0, and 22.0 mL/L of a stock solution. All assays were done in triplicate for each group and a control group. Fish were fasted throughout the experimental time and maintained at density of 1 g/L, according to Cetesb [9] methodology.

Dissolved oxygen, pH, and electrical conductivity of water were daily monitored (YSI 556 MPS-Multiprobe System); water temperature was maintained at 22.8°C. Throughout the experiment, fish were evaluated daily by direct observation, looking for signs of intoxication, like changes in behavior, macroscopic external lesions, location in aquarium, opercular movements, erratic swimming, and mortality.

### 2.4. Statistical Analysis

The quantitative determination peaks areas in the sample chromatograms was performed using the regression equations from the CLA, CFA, and RUT calibration curves [7]. The calculations of the levels of OA, QAD, and LMP were performed using the equation *A* = (1/2)*h* · *W*_*b*_ corresponding to calculate the area of a chromatographic peak [7].

Measurements of general toxicity obtained during the first 24 hours of exposure to the extract were analyzed by Trimmed Spearman-Karber method. The mortality results were analyzed considering each aquarium as an experimental unit, expressing the mortality percentage and the number of deaths in each concentration.

A correlation between concentration and mortality was analyzed by the method of Pearson. The water quality parameters were analyzed by ANOVA using the statistical package Statistical Analysis System (SAS) v.8.0.

## 3. Results

**Figure 1:**
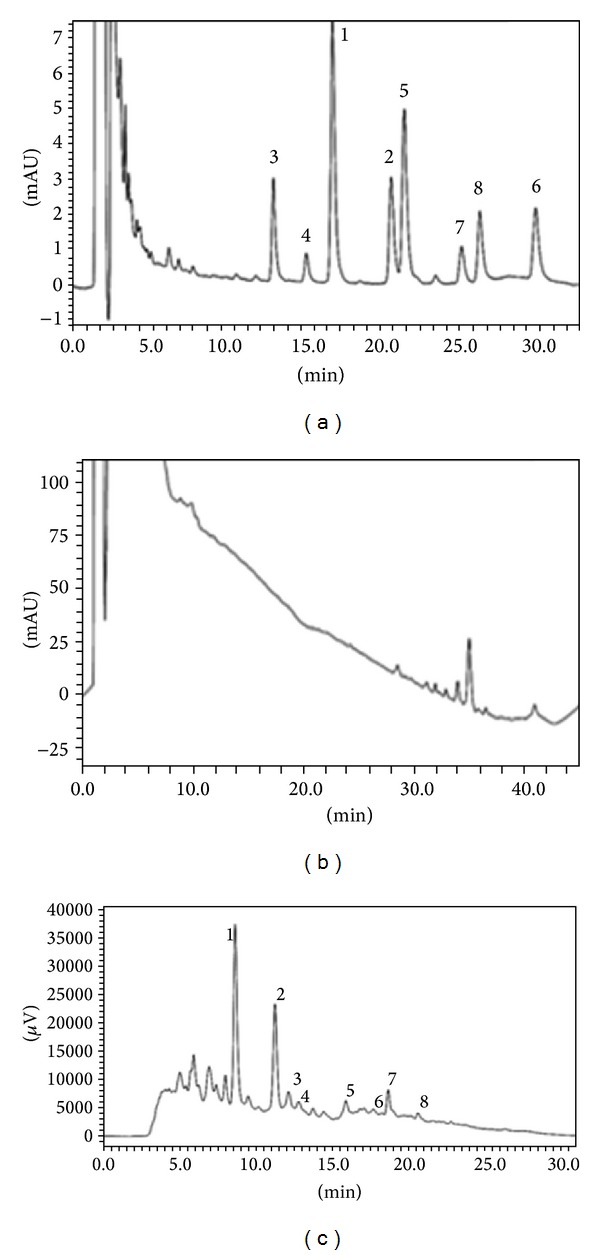
Chromatographic profile of aqueous extract of *Uncaria tomentosa* at 245, 245, and 325 nm, respectively. (a) OA (6), (b) QAD (7), and (c) LMP (5).

**Figure 2:**
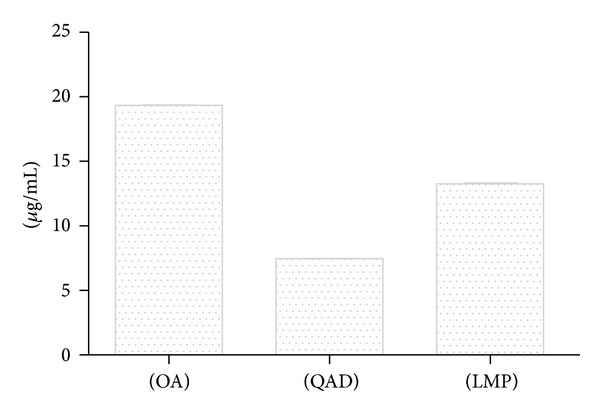
Concentrations of derivatives of oxindole alkaloids (OA), quinovic acid derivatives (QAD), and low molecular weight polyphenols present in the aqueous extract.

The HPLC-PDA analysis of aqueous extract of *U. tomentosa* bark revealed three characteristic groups of compounds of this plant, thereby confirming with certainty the identity of the sample. These compounds were oxindole alkaloids (OA), quinovic acid derivatives (QAD), and low molecular weight polyphenols (LMP). OA were the most abundant (19.66 ± 0.01 *μ*g/mL), followed by LMP (13.25 ± 0.07 *μ*g/mL) and finally QAD (7 : 46 ± 0 : 01 *μ*g/mL) (Figures 1 and 2).

**Figure 3:**
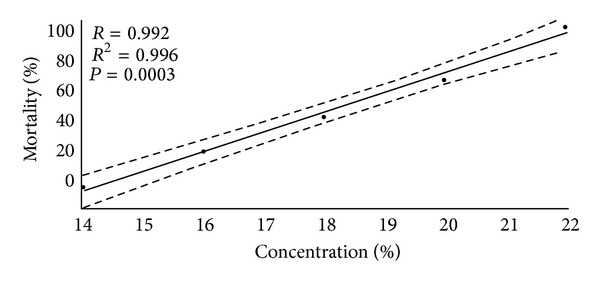
Correlation between the concentration of the extract and the mortality of animals.

It is clear from Figure 3 that, as concentration of the extract increased, fish mortality also increased (*R* = 0.992), which indicates a direct proportional relationship between mortality, and concentration of *U. tomentosa*, reaching 100% at the highest concentration.

In the final test, mortality at 14.0 mL/L was not observed, unlike at 22.0 mL/L where 100% mortality was observed. As a result it was found that the estimated value of 48 h LC50 of aqueous extract of *U. tomentosa* bark for *H. eques* is 18.16 mL/L, with a lower limit of 17.10 mL/L and a higher one of 19.27 mL/L.

The physiochemical characteristics of test water are listed in Table 1. The temperature and the electrical conductivity were stable throughout the experiment. Dissolved oxygen decreased when the concentration of the extract was higher, ranging from 6.3 to 2.2. The pH of the control group was higher than the other treatments (8.3 to 7.5 ± 0.17) probably due to the presence of organic matter that reacts with water forming carbonic acid.

## 4. Discussion

Plants have been used, empirically, for centuries for medicinal purposes. Currently, it requires the identification and quantification of the active principles, in order to preserve their chemical and pharmacological characteristics, ensuring their biological action, clinical safety, and enhancement of their therapeutic potential. Thus, it was verified by the use of high-performance liquid chromatography (HPLC) that the active principles were preserved after a preparation of an aqueous extract of *Uncaria tomentosa* bark, in agreement with the results obtained by Montoro et al. [10] for oxindole alkaloids, Pavei et al. [7] for quinovic acid derivatives, and Pavei et al. [5] for low molecular weight polyphenols.

Keplinger et al. [1] have reported low toxicity of this plant in rats. There are no studies about *U. tomentosa* toxicity in fish or any other aquatic animal. It is noteworthy that fish are especially sensitive to toxins due in part to their type of respiration and high perfusion with environment.

Based on the classification of Zucker [11] the aqueous extract of *U. tomentosa* bark is considered as “practically nontoxic” for Jewel tetra. This author considers the values of LC50 below 100 mg/L as practically nontoxic for fish and aquatic invertebrates. On the other hand, there are reports of other herbal extracts fairly used in fish with similar LC50 and considered safe as aqueous extract of neem (*Azadirachta indica*), tested in Nile tilapia with LC50 of 2740 mg/L [12], and aqueous extract of *Terminalia catappa* tested in *Phalloceros caudimaculatus, *which presented a LC50 of 208.52 mg/L [13].

Though pH influences the toxicity due to an electrolyte imbalance, does not appear to have occurred in this study. Mortality was not attributed to pH; even at high concentrations of the extract, the values of pH were similar to control group. It is possible that the cause of the mortality is associated with the low levels of dissolved oxygen which was inversely proportional to the increased extract concentration [14].

In all groups containing *U. tomentosa* clinical and behavioral alterations characteristic of hypoventilation were observed, such as increase of opercular movements and fish swimming at surface. This condition worsened with increasing extract concentration and exposure time. This can explained due to the decomposition of organic matter extract, which requires oxygen present in the water. Furthermore, it is possible that the excess of extract has been accumulated in the gills, reducing gaseous and ionic exchanges [13].

On the other hand, it is likely that a decrease of physiological compensatory mechanisms happened due to receptor blockage, resulting in a competitive interaction between chemical compounds of the extract by O_2_ chemoreceptors that are located on gill arches [13]. In addition, polyphenolic compounds soluble in polar organic solvents are capable of precipitating proteins [11], leading to accumulation of these compounds and worsening the clinical situation.

A determining factor seems to have been the interaction of chemical derivates of oxindole alkaloids with dopamine, leading to depression of the central nervous system and changes in blood pressure resulting in reduced cardiac frequency in mice [15].

Fish presented clinical and behavioral alterations after 24 hours of exposure to the plant extract with concentration above 20.0 mL/L of extract, being compatible with a dopamine interaction case.

It was concluded that the preparation of the aqueous extract of *U. tomentosa* bark did not substantially affect the chemical components of the plant. Oxindole alkaloids, quinovic acid derivatives, and low molecular weight polyphenols were maintained at adequate levels. *U. tomentosa* LC50 after 48 h in *H. eques* was 1816 mg/L; however, concentrations of active ingredients in commercial extracts of *U. tomentosa* vary widely from different manufacturers. Therefore, the use of this plant in fish was considered “practically nontoxic” to Jewel fish. Nonetheless, at high concentrations an interaction with dopamine is possible. The use of this plant in fish is safe enough for medical and prophylactic purposes as those used in mammals.

## Figures and Tables

**Table 1:** Physical-chemical parameters of water.

Parameters	Concentration					
	0	14	16	18	20	22
Temperature (°C)	22.7	22.8	22.8	22.8	22.7	22.8
Conductivity (uS)	175.2	185.4	184.2	189.5	190.1	190.2
Dissolved oxygen (mL/L)	6.3	2.6	2.9	2.4	2.4	2.2
pH	8.3	7.7	7.7	7.5	7.3	7.4
